# Statins, HMG-CoA Reductase Inhibitors, Improve Neovascularization by Increasing the Expression Density of CXCR4 in Endothelial Progenitor Cells

**DOI:** 10.1371/journal.pone.0136405

**Published:** 2015-08-26

**Authors:** Kuang-Hsing Chiang, Wan-Li Cheng, Chun-Ming Shih, Yi-Wen Lin, Nai-Wen Tsao, Yung-Ta Kao, Chih-Ting Lin, Shinn-Chih Wu, Chun-Yao Huang, Feng-Yen Lin

**Affiliations:** 1 Division of Cardiology and Cardiovascular Research Center, Department of Internal Medicine, Taipei Medical University Hospital, Taipei, Taiwan; 2 Cardiovascular Research Center, National Yang-Ming University, Taipei, Taiwan; 3 Graduate Institute of Biomedical Electronics and Bioinformatics, National Taiwan University, Taipei, Taiwan; 4 Department of Internal Medicine, School of Medicine, College of Medicine, Taipei Medical University, Taipei, Taiwan; 5 Institute of Oral Biology, National Yang-Ming University, Taipei, Taiwan; 6 Division of Cardiovascular Surgery, Department of Surgery, Taipei Medical University Hospital, Taipei, Taiwan; 7 Department of Animal Science and Technology, National Taiwan University, Taipei, Taiwan; University of Illinois at Chicago, UNITED STATES

## Abstract

Statins, inhibitors of 3-hydroxy-3-methylglutaryl-coenzyme A (HMG-CoA) reductase, are used to reduce cholesterol biosynthesis in the liver. Accordingly, statins regulate nitric oxide (NO) and glutamate metabolism, inflammation, angiogenesis, immunity and endothelial progenitor cells (EPCs) functions. The function of EPCs are regulated by stromal cell-derived factor 1 (SDF-1), vascular endothelial growth factor (VEGF), and transforming growth factor β (TGF-β), etc. Even though the pharmacologic mechanisms by which statins affect the neovasculogenesis of circulating EPCs, it is still unknown whether statins affect the EPCs function through the regulation of CXCR4, a SDF-1 receptor expression. Therefore, we desired to explore the effects of statins on CXCR4 expression in EPC-mediated neovascularization by *in vitro* and *in vivo* analyses. In animal studies, we analyzed the effects of atorvastatin or rosuvastatin treatments in recovery of capillary density and blood flow, the expression of vWF and CXCR4 at ischemia sites in hindlimb ischemia ICR mice. Additionally, we analyzed whether the atorvastatin or rosuvastatin treatments increased the mobilization, homing, and CXCR4 expression of EPCs in hindlimb ischemia ICR mice that underwent bone marrow transplantation. The results indicated that statins treatment led to significantly more CXCR4-positive endothelial progenitor cells incorporated into ischemic sites and in the blood compared with control mice. *In vivo*, we isolated human EPCs and analyzed the effect of statins treatment on the vasculogenic ability of EPCs and the expression of CXCR4. Compared with the control groups, the neovascularization ability of EPCs was significantly improved in the atorvastatin or rosuvastatin group; this improvement was dependent on CXCR4 up-regulation. The efficacy of statins on improving EPC neovascularization was related to the SDF-1α/CXCR4 axis and might be regulated by the NO. In conclusion, atorvastatin and rosuvastatin improved neovascularization in hindlimb ischemia mice; this effect may have been mediated by increased CXCR4 expression in EPCs.

## Introduction

There are numerous diseases that can cause endothelial cell damage and atherosclerosis progression, including smoking, diabetes mellitus, dyslipidemia and hypertension. Particularly, dyslipidemia is the most important risk factor for atherosclerosis [1]. Endothelial dysfunction is also a systemic disorder and a key variable in the progression of the atherosclerosis [2]. Current evidence suggests that endothelial repair is driven not only by local cell but also by the contribution of circulating cells [3]. Circulating cells with the ability to repair the endothelium are named endothelial progenitor cells (EPCs). In patients with atherosclerosis, the numbers and activity of the EPCs were decreased [4]. The repair abilities of EPCs are affected by lifestyle risk factors and by pharmacological therapies [5]. Thus, the numbers and activities of circulating EPCs are thought to be important determinants of cardiovascular disease [6]. A great deal of evidence has suggested that the management of SDF-1 increases blood flow and perfusion via the recruitment of EPCs. The stromal cell-derived factor 1/C-X-C chemokine receptor type 4 (SDF-1/CXCR4) axis plays an important role in angiogenesis by recruiting EPCs from the bone marrow [7]. Previous studies have demonstrated that the phosphoinositide 3-kinase/AKT/eNOS signal transduction pathway is involved in the SDF-1/CXCR4 axis-mediated migration of EPCs [8]. Conclusively, it is clinically important to estimate the bioactivity of EPCs and to find an appropriate intervention for improving EPC function [6].

Owing to the inhibitor of HMG-CoA reductase, the pleiotropic effects of statins have been demonstrated. Statins treatment has been demonstrated to enhance EPC functions such as mobilization, proliferation, migration, adhesion, and differentiation [9]. Statin administration has been revealed to stimulate angiogenesis by up-regulating endothelial nitric oxide synthase (eNOS) [10], which plays an important role in the mobilization of bone marrow EPCs [11,12]. Among the current clinically used statins, atorvastatin and rosuvastatin have the most potency [13]. Short-term treatment with rosuvastatin significantly increased the number of EPCs in patients with heart failure compared with healthy controls [14]. Atorvastatin therapy increased the migration and adhesion of EPCs in patients with chronic pulmonary heart disease. Nevertheless, the pharmacologic mechanisms of actions of atorvastatin and rosuvastatin on EPC neovasculogenesis await further study.

In this study, we explored the mechanisms of atorvastatin and rosuvastatin on EPC-mediated neovascularization by *in vitro* and *in vivo* analyses. Furthermore, we analyzed the mechanism of how statins affect the abilities of EPCs.

## Materials and Methods

### Animal grouping and statin administration

Male ICR mice were purchased from BioLASCO Taiwan Co., Ltd. (Yi-Lan, Taiwan). Male eGFP transgenic mice (ICR background) were a kind gift from Dr. Shinn-Chih Wu. All animals were treated according to protocols approved by the Institutional Animal Care and Use Committee (IACUC) of the Taipei Medical University, Taipei, Taiwan (admission No.: LAC-2014-0155). The experimental procedures and animal care conformed to the “Guide for the Care and Use of Laboratory Animals” published by the U.S. National Institutes of Health (NIH Publication No. 85–23, revised 1996). Mice were fed a chow diet (Scientific Diet Services, Essex, UK). Forty-five male ICR mice (6–8 weeks old) and five male eGFP transgenic mice were used, and the animals were divided into nine groups. Group 1 (naïve control) was the naïve control group; group 2 [ischemia (IS)] received a hindlimb ischemia operation at week 1; group 3 [IS+atorvastatin 2 mg/kg body weight (BW)] received a hindlimb ischemia operation at week 1 and oral administration of 2 mg/kg BW/day atorvastatin throughout the experiment (4 weeks); group 4 (IS+atorvastatin 8 mg/kg BW) received a hindlimb ischemia operation and oral gavage of 8 mg/kg BW/day atorvastatin once per day throughout the experiment (4 weeks); group 5 (IS+rosuvastatin 2 mg/kg BW) received a hindlimb ischemia operation and oral gavage of 2 mg/kg BW/day rosuvastatin once per day throughout the experiment (4 weeks); group 6 (IS+rosuvastatin 4 mg/kg BW) received a hindlimb ischemia operation and oral gavage of 4 mg/kg BW/day rosuvastatin once per day throughout the experiment (4 weeks); group 7 (IS+BMT) received a hindlimb ischemia operation and received an injection of bone marrow cells at week 1; group 8 (BMT+IS+ atorvastatin 8 mg/kg BW) received bone marrow cells injection at week 1, and received a hindlimb ischemia operation at week 6, as well as oral gavage of 2 mg/kg BW/day rosuvastatin once per day after ischemic surgery throughout the experiment; group 9 (BMT+IS+rosuvastatin 4 mg/kg BW) received an injection of bone marrow cells at week 1 and received a hindlimb ischemia operation at week 6, as well as oral gavage of 2 mg/kg BW/day rosuvastatin once per day after ischemic surgery throughout the experiment.

### Mouse hindlimb ischemia model

At the beginning of experimentation, unilateral hindlimb ischemia was induced in the mice by ligating and excising the right femoral artery, as previously described [15]. Briefly, the animals were anesthetized by an intraperitoneal injection of Xylocaine (2 mg/kg of BW) plus Zoletil (containing the dissociative anesthetic Tiletamine/Zolazepam at a ratio of 1:1; 5 mg/kg of BW). The proximal and distal portions of the femoral artery were ligated with a silk thread, and the blood vessel was cut by approximately 0.2 centimeters. Hindlimb blood perfusion was measured with a laser Doppler perfusion imager system (Moor Instruments Limited, Devon, UK) before and after the surgery and was then followed on a weekly basis. The animals were sacrificed by cervical dislocation without sedation at the end of the seven experimental weeks. To avoid the influence of ambient light and temperature, the results are expressed as the ratio of perfusion in the right (ischemic) versus left (non-ischemic) limb.

### Mouse bone marrow transplantation

Bone marrow transplantation was performed as previously described [15]. Recipient wild-type ICR mice (BLTW: CD1 strain) were lethally irradiated with a total dose of 9.0 Gy at 8 weeks of age. eGFP transgenic mice (ICR background) that ubiquitously expressed enhanced GFP were used as the donors. After being irradiated, each recipient mouse received unfractionated bone marrow cells (5×10^6^) from an eGFP mouse via tail vein injection. Six weeks after bone marrow transplantation, the chimeric mice underwent unilateral hindlimb ischemia surgery and statin treatment. Repopulation by eGFP-positive bone marrow cells was determined to be 95%, as measured by flow cytometry. Four weeks after the induction of hindlimb ischemia, ischemic thigh muscles were harvested for histological analysis. Bone marrow-derived EPCs were stained with antibodies directed against eGFP (Millipore, Billerica, MA, USA) and CD34 (Becton Dickinson, Franklin Lakes, NJ, USA). The EPC density was determined by counting eGFP/CD34 double-positive cells (yellow color) using a fluorescence microscope at a magnification of 400x.

### Immunohistochemical staining for morphometry

Whole ischemic limbs were harvested, the adhering tissues and femora were carefully removed, and the samples were immersion-fixed with 4% buffered paraformaldehyde. The staining was performed on serial 5-μm-thick paraffin-embedded sections. Immunohistochemical staining was performed on mouse ischemic thigh muscles (sartorius muscle, gracilis muscle, adductor muscle and semimembranosus muscle were included) using goat anti-Von Willebrand factor (vWF; Santa Cruz Biotechnologies, San Jose, CA, USA), rabbit anti-CD34 (Millipore, Billerica, MA, USA), and rabbit anti-CXCR4 (Abcam, San Francisco, CA, USA) antibodies, followed by counterstaining with Hoechst. The stained slides were observed using fluorescence microscopy. Three cross-sections were analyzed for each animal. Ten different fields from each tissue preparation were randomly selected, and visible capillaries were counted. The densities of capillaries and CXCR4 expression were expressed as the fluorescence/myofiber ratio.

### Flow cytometry

To investigate the effects of statins on mobilization and CXCR4 expression in circulating EPCs in response to tissue ischemia, a fluorescence-activated cell sorting (FACS) Caliber flow cytometer (Becton Dickinson, San Jose, CA, USA) was used. A volume of 300 μL peripheral blood was incubated with fluorescein isothiocyanate (FITC) anti-mouse CD34 (eBioscience, San Diego, CA, USA), phycoerythrin (PE) anti-mouse Flk-1 (VEGFR-2, eBioscience, San Diego, CA, USA), and rat anti-mouse CXCR4 (Becton Dickinson, San Jose, CA, USA) antibodies. Isotype-identical antibodies served as controls (Becton Dickinson, Franklin Lakes, NJ, USA). After incubation for 30 min, the cells were lysed (PharmLyse; Becton Dickinson, Franklin Lakes, NJ, USA), washed with phosphate-buffered saline (PBS), and fixed in 2% paraformaldehyde before analysis. Each analysis included 30,000 events. Circulating EPCs were considered to be from the mononuclear cell population and were gated by double-positive staining for CD34 and Flk-1. Additionally, the percentage of CXCR4-expressing EPCs was displayed as the ratio of CXCR4-positive cells to CD34-Flk-1-double-positive cells.

### Cultivation of human EPCs

Total mononuclear cells (MNCs) were isolated from 40 ml peripheral blood from healthy young male volunteers by density-gradient centrifugation with Histopaq-1077 (density 1.077 g/mL; Sigma-Aldrich, CA, USA). The Taipei Medical University-Institutional Review Board approved this study (IRB No.: CRC-04-10-04 and No.: 201302008), and all participants provided their written informed consent to participate in this study. MNCs (1x10^7^ cells) were plated in 2 ml endothelial growth medium (EGM-2 MV; Cambrex, Charles, IA, USA) with supplements (hydrocortisone, R3-insulin-like growth factor 1, human vascular endothelial growth factor, human fibroblast growth factor, gentamicin, amphotericin B, vitamin C, and 20% fetal bovine serum) on fibronectin-coated six-well plates at 37°C in a 5% CO_2_ incubator. The cultures were observed daily, and after 4 days of culture, the media were changed, and nonadherent cells were removed. Attached early EPCs were elongated, with a spindle shape. Thereafter, media were replaced every 3 days, and each colony/cluster was observed. A certain number of early EPCs continued to grow into colonies of late EPCs, which emerged 2–4 weeks after the start of MNC culture. The characterization of EPCs was conducted as previously described [16].

### Tube formation assay

The tube formation assay was performed on EPCs to assess their angiogenic capacity, which is involved in new vessel formation [17]. The *in vitro* tube formation assay was performed using the Angiogenesis Assay Kit (Millipore, Billerica, MA, USA) according to the manufacturer’s protocol. In brief, the ECMatrix gel solution was thawed at 4°C overnight, mixed with the ECMatrix diluent buffer, and placed in a 96-well plate at 37°C for 1 hour to allow the matrix solution to solidify. EPCs were treated with or without 2.5–10 μM of atorvastatin or rosuvastatin for 24 hours and then harvested. A total of 10^4^ cells were placed on the matrix solution with 10 ng/mL recombinant human stromal cell-derived factor 1 (SDF-1), and the samples were incubated at 37°C for 12 hours. The naïve EPCs group was used as the negative control. Tube formation was inspected under an inverted light microscope. Images of four representative fields were taken, and the averages of the branching points formed by the cells were quantified.

### Migration assay

The migratory function of late EPCs, which is essential for vasculogenesis, was evaluated by a modified Boyden transwell chamber (Costar, Hanover Park, IL, USA) assay [17]. Late EPCs were incubated with 10 μM atorvastatin or rosuvastatin for 24 h. Then, 4x10^4^ treated EPCs were placed in the upper chamber of 24-well Transwell plates with polycarbonate membranes (8 μm pores) in EGM-2 MV medium. EGM-2 MV medium with VEGF (50 ng/mL) and SDF-1 (10 ng/mL) was placed in the lower chamber. After incubation for 24 h, the membranes were washed briefly with PBS and fixed with 4% paraformaldehyde. The upper sides of the membranes were wiped gently with a cotton ball. The membranes were then stained using hematoxylin solution and removed. The magnitude of late EPC migration was evaluated by counting the migrated cells in six random high-power (100x) microscope fields.

### Quantitative real-time polymerase chain reactions

Total RNA was isolated using a TRIzol reagent kit (Invitrogen, Carlsbad, CA, USA) according to the manufacturer’s instructions. cDNA was synthesized from the total RNA using Superscript II reverse transcriptase. Quantitative real-time PCR was performed using a FastStart DNA Master SYBR Green I kit and a LightCycler (Roche, CA, USA) with 35 cycles for GAPDH, eNOS and CXCR4. The mRNA levels of eNOS and CXCR4 were determined in arbitrary units by comparison with an external DNA standard. Glyceraldehyde 3-phosphate dehydrogenase (GAPDH) was amplified in the same samples to verify RNA abundance. All of the specific primers were synthesized by Medclub Scientific Co., LTD. (Taoyuan, Taiwan). The PCR primers used for amplification of GAPDH, eNOS and CXCR4 are as follows: GAPDH, 5’-tgccccctctgctgatgcc-3’ and 5’-cctccgacgcctgcttcaccac-3’; eNOS, 5’-agacctttaaagaagtggccaacg-3’ and 5’-catactcatccatacacaggaccc-3’; CXCR4, 5’-cactgtgcacaagtggatttccat-3’ and 5’-tggaaacagatgaatgtccacctc-3’.

### Western blotting analysis of mouse skeletal muscle and human EPCs

Briefly, 0.5 g of mouse skeletal muscle or human EPCs was lysed in lysis buffer (20 mM Tris-HCl (pH 7.5), 150 mM NaCl, 1 mM Na_2_EDTA, 1 mM EGTA, 1% Triton, 2.5 mM sodium pyrophosphate, 1 mM beta-glycerophosphate, 1 mM Na_3_VO_4_, 1 μg/mL leupeptin) (catalog: 9803, Cell Signaling, San Diego, CA, USA). The protein lysates were subjected to SDS-PAGE followed by transfer onto a PVDF membrane. The membranes were probed with monoclonal antibodies against total eNOS (Cell Signaling, San Diego, CA, USA), phospho-eNOS (Cell Signaling, San Diego, CA, USA), and β-actin (Millipore, Billerica, MA, USA). Bands were visualized using chemiluminescent detection reagents.

### Electron spin resonance spectroscopy (ESRS)

The spin-probe colloid Fe(DETC)2 (Noxygen) was used as a probe to detect the NO production in EPCs; this was performed as previously described [18,19]. In brief, 200 μL EPC-cultured medium was mixed with 400 μL colloid Fe(DETC)2, followed by incubation for 1 hr at 37°C. The samples were recorded on n ESRS (model: EMX-6/1, Bruker BioSpin, San Antonio, TX, USA) with the following instrumental settings: center field 3295.0 G, sweep width 100.0 G, static field 3415.0 G, microwave power 2.0 mW, microwave frequency 9.8 GHz, modulation amplitude 10.0 G, modulation frequency 100.0 GHz, resolution in X 1024 points, sweep time 10.5 seconds and number of X-scans 5.

### Statistical analyses

For *in vitro* and animal study,values are expressed as the mean ± SEM. Statistical evaluation was performed using Student’s t test and one- or two-way ANOVA followed by Dunnett’s test. A probability value of p<0.05 was considered significant.

## Results

### Atorvastatin and rosuvastatin enhanced the recovery of capillary density in ischemic hindlimb mice

**Fig 1:**
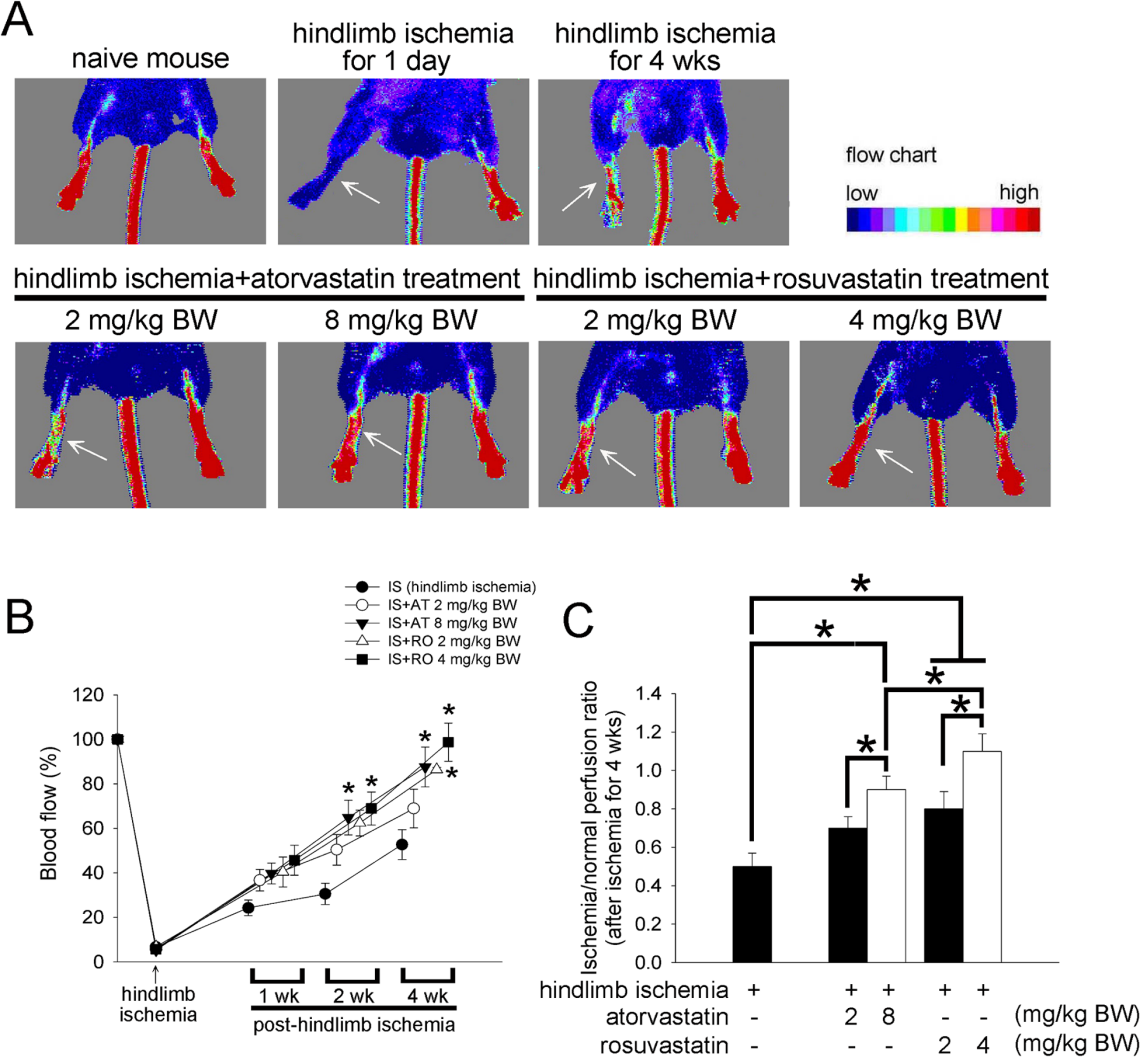
The effect of atorvastatin and rosuvastatin on blood flow recovery after hindlimb ischemia in ICR mice. (A) Upper column, representative results of laser Doppler measurements before the operation (control), and 1 day or 4 weeks after hindlimb ischemia surgery; lower column, representative results of laser Doppler measurements 4 weeks after hindlimb ischemia surgery in mice treated with 2 and 8 mg/kg BW of atorvastatin or 2 and 4 mg/kg BW of rosuvastatin. Color scale illustrates blood flow variations from minimal (dark blue) to maximal (red) values. Arrows indicate ischemic (right) limb after hindlimb ischemia surgery. (B) Doppler perfusion ratios (ischemic/non-ischemic hindlimbs) over time in the different groups. Administration of 8 (▼) mg/kg BW atorvastatin and 4 (■) mg/kg BW rosuvastatin for 2 weeks enhanced beneficial blood flow recovery compared with the non-administered (●) group 2 weeks after hindlimb ischemia surgery. Additionally, the low dose of rosuvastatin (△; 2 mg/kg BW) enhanced beneficial blood flow recovery at week 4 after surgery. There was no significant difference in blood flow in the limbs in the 2 mg/kg BW atorvastatin-treated group (○) compared with the non-administered group. (C) Four weeks after ischemic surgery, the ischemia/normal perfusion ratios in the 8 mg/kg BW atorvastatin and 2 mg/kg BW and 4 mg/kg BW rosuvastatin-treated groups were higher than the ratio in the non-statin-treated group. The results are expressed as the mean ± SEM (n = 5, *p < 0.05 was considered to be significant).

To evaluate the angiogenic effects of atorvastatin and rosuvastatin, we performing unilateral hindlimb ischemia surgery on wild-type male ICR mice (*n* = 5 for each group). Ligation and excision of the femoral artery effectively blocked blood flow in ICR mice (Fig 1A). Blood flow in ischemic limbs was significantly recovered in the 8 mg/kg BW atorvastatin-treated mice or 4 mg/kg BW rosuvastatin-treated mice compared with the controls when the treatments were started the second week (Fig 1B). After four weeks of the treatments, daily administration of 8 mg/kg BW atorvastatin or 2 and 4 mg/kg BW rosuvastatin also significantly improved the blood flow (Fig 1B). Four weeks after statin administration, the ischemia/normal perfusion ratio in the 8 mg/kg BW atorvastatin or 2 and 4 mg/kg BW rosuvastatin-treated group was higher than that in the control group (Fig 1C). These results indicated that atorvastatin or rosuvastatin treatment enhanced the recovery of capillary density after hindlimb ischemia in ICR mice. Thus, the statin-treated mice had accelerated blood flow recovery after surgery compared with the controls.

### Atorvastatin and rosuvastatin promoted small vessel formation

**Fig 2:**
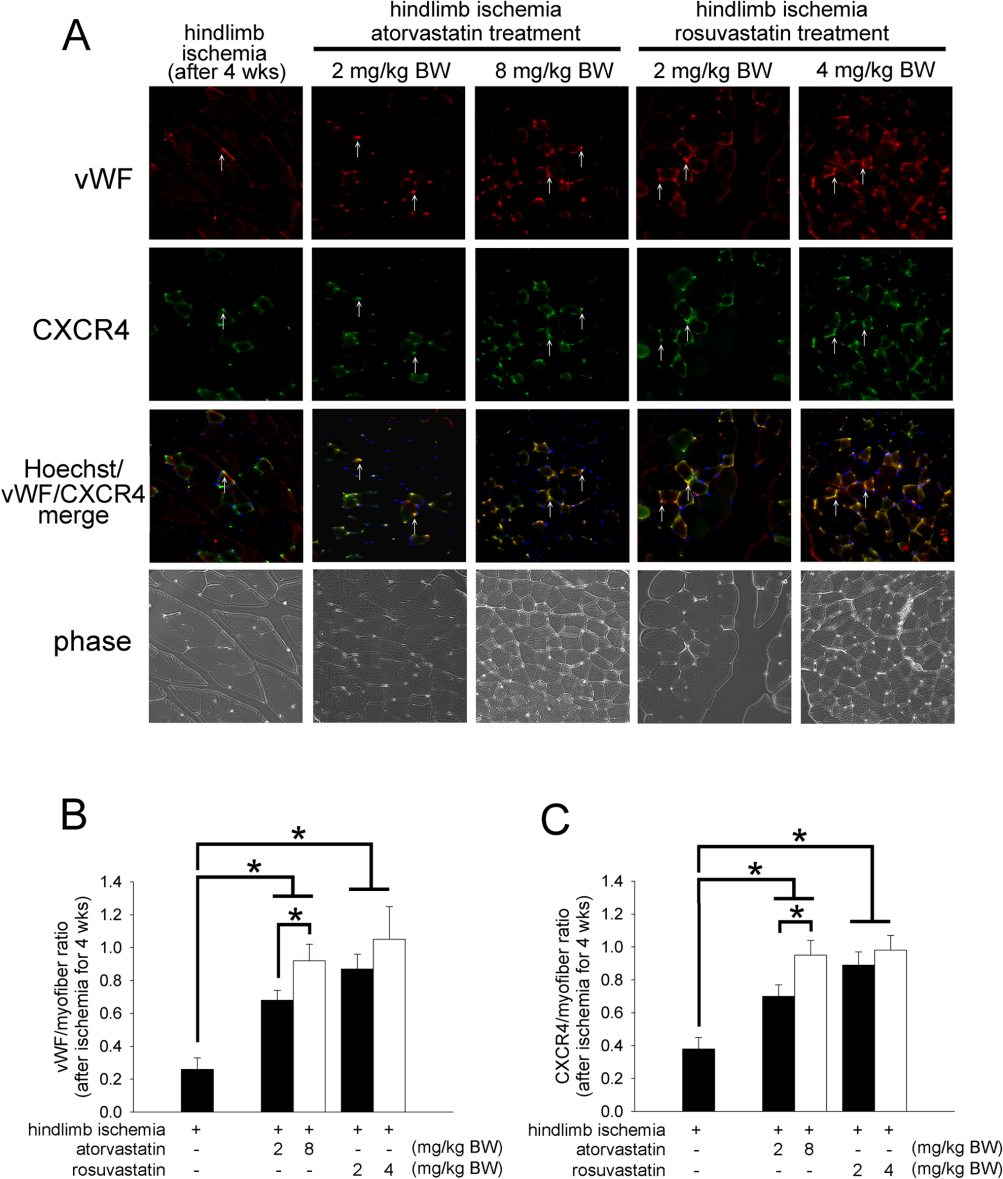
The effects of atorvastatin and rosuvastatin on vWF and CXCR4 expression in the skeletal muscle after hindlimb ischemia in ICR mice. (A) Mice were sacrificed 4 weeks after surgery, and the number of capillaries and expression of CXCR4 in the ischemic muscles were visualized by anti-vWF or anti-CXCR4 immunostaining (original magnification x400), respectively. The vWF and CXCR4 are indicated with white arrows. (B) The graph shows the quantification of the capillary density in hindlimb ischemic and statin-administered mice. (C) The graph shows the quantification of the CXCR4 density in hindlimb ischemic and statin-administered mice. The results are expressed as the mean ± SEM (n = 5, *p < 0.05 was considered to be significant).

To extend our observations, we analyzed the blood vessel densities in ischemic muscle tissues. The expression of von Willebrand factor (vWF) protein, a critical marker of endothelial cells in small blood vessels, was analyzed in the ischemic sites (Fig 2A). Four weeks after surgery, atorvastatin or rosuvastatin administration significantly increased the number of capillaries in the ischemic muscle compared with that in the control (Fig 2A and 2B). To determine whether statins are involved in SDF-1/CXCR4 axis-mediated neovasculogenesis, we analyzed the expression of CXCR4 in the ischemic muscle tissue. We also quantified the expression density of CXCR4 in atorvastatin-, rosuvastatin- or untreated hindlimb ischemia mice (Fig 2C). The expression densities of CXCR4 in the 2 and 8 mg/kg BW atorvastatin-treated and 2 and 4 mg/kg BW rosuvastatin-treated mice were higher than those in the controls. Above all, we found that both atorvastatin and rosuvastatin could improve the recovery rate of capillaries after ischemia and up-regulate CXCR4 expression in ischemic tissues.

### Atorvastatin and rosuvastatin increased the homing of CXCR4^+^ EPCs at the ischemic sites

**Fig 3:**
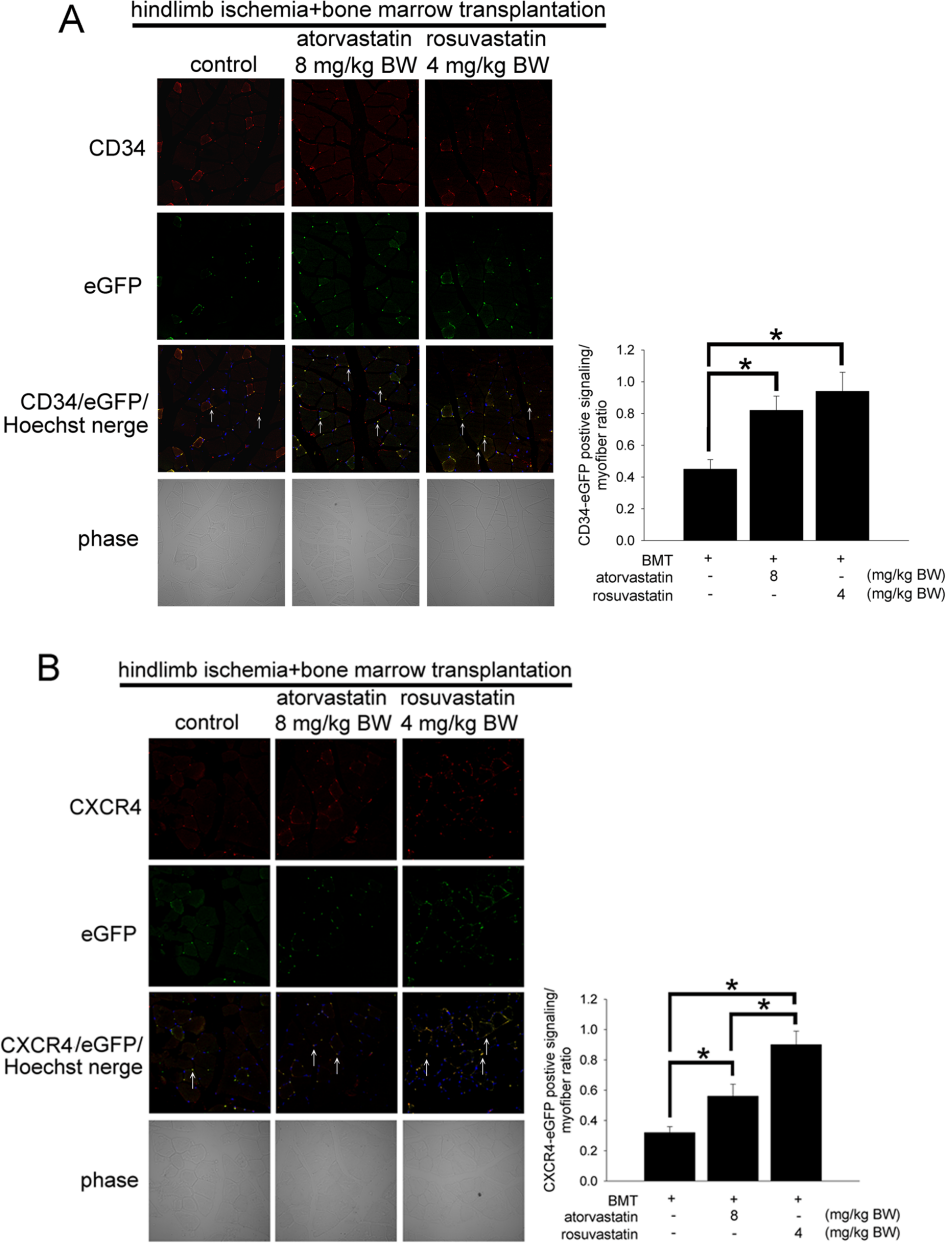
The effects of atorvastatin and rosuvastatin on EPC homing in hindlimb ischemic mice. (A) Hindlimb ischemia was induced in ICR mice that received eGFP-labeled mouse bone marrow cells. Immunostaining of the ischemic hindlimb muscle with anti-CD34-conjugated Alexa 633 (red) antibody in ICR mice treated with 8 mg/kg BW atorvastatin or 4 mg/kg BW rosuvastatin. The CD34-positive homed hematopoietic stem precursor cells are indicated with white arrows. The bar graph shows the eGFP-CD34-double positive signaling/myofiber ratio. (B) Immunostaining of ischemic hindlimb muscle with anti-CXCR4 conjugated Alexa 633 (red) antibody in ICR mice treated with statins. CXCR4-positive EPCs are indicated with white arrows. Hoechst stain was used to counterstain the nucleus. The ischemic hindlimb tissue was evaluated by fluorescence microscopy at a magnification of 400x. The bar graph shows the eGFP-CXCR4-double positive signaling/myofiber ratio. The results are expressed as the mean ± SEM (n = 5, *p < 0.05 was considered to be significant).

Hindlimb ischemia mice received eGFP mouse bone marrow cells, and the levels of CD34^+^/eGFP^+^ cells were determined to investigate the effects of atorvastatin or rosuvastatin on EPC mobilization. After 4 weeks of statins treatment, the atorvastatin- and rosuvastatin-treated mice had higher numbers of CD34^+^/eGFP^+^ cells than the control mice. Treatment with 8 mg/kg BW atorvastatin or 4 mg/kg BW rosuvastatin significantly up-regulated the mobilization of EPCs to ischemic hindlimb tissues (Fig 3A). These results indicated that atorvastatin or rosuvastatin treatment increased the numbers of circulating EPCs in the hindlimb tissues. To clarify whether atorvastatin and rosuvastatin was involved in SDF-1/CXCR4 axis-mediated neovasculogenesis by EPCs, we analyzed the expression of CXCR4 in the EPCs after statins treatments. CXCR4 expression was detected in the ischemic hindlimb muscle 4 weeks after surgery. Our results indicate that treatment with 8 mg/kg BW atorvastatin or 4 mg/kg BW rosuvastatin increased the numbers of CXCR4^+^ EPCs in ischemic hindlimb muscles (Fig 3B). The bar graph shows that eGFP^+^/CXCR4^+^ EPCs were significantly more abundant after atorvastatin or rosuvastatin treatment. Above all, our results indicated that statins increased the migration of the CXCR4^+^ EPCs to the ischemic tissues.

### Atorvastatin and rosuvastatin increased the intensity of CXCR4 expression on circulating EPCs

**Fig 4:**
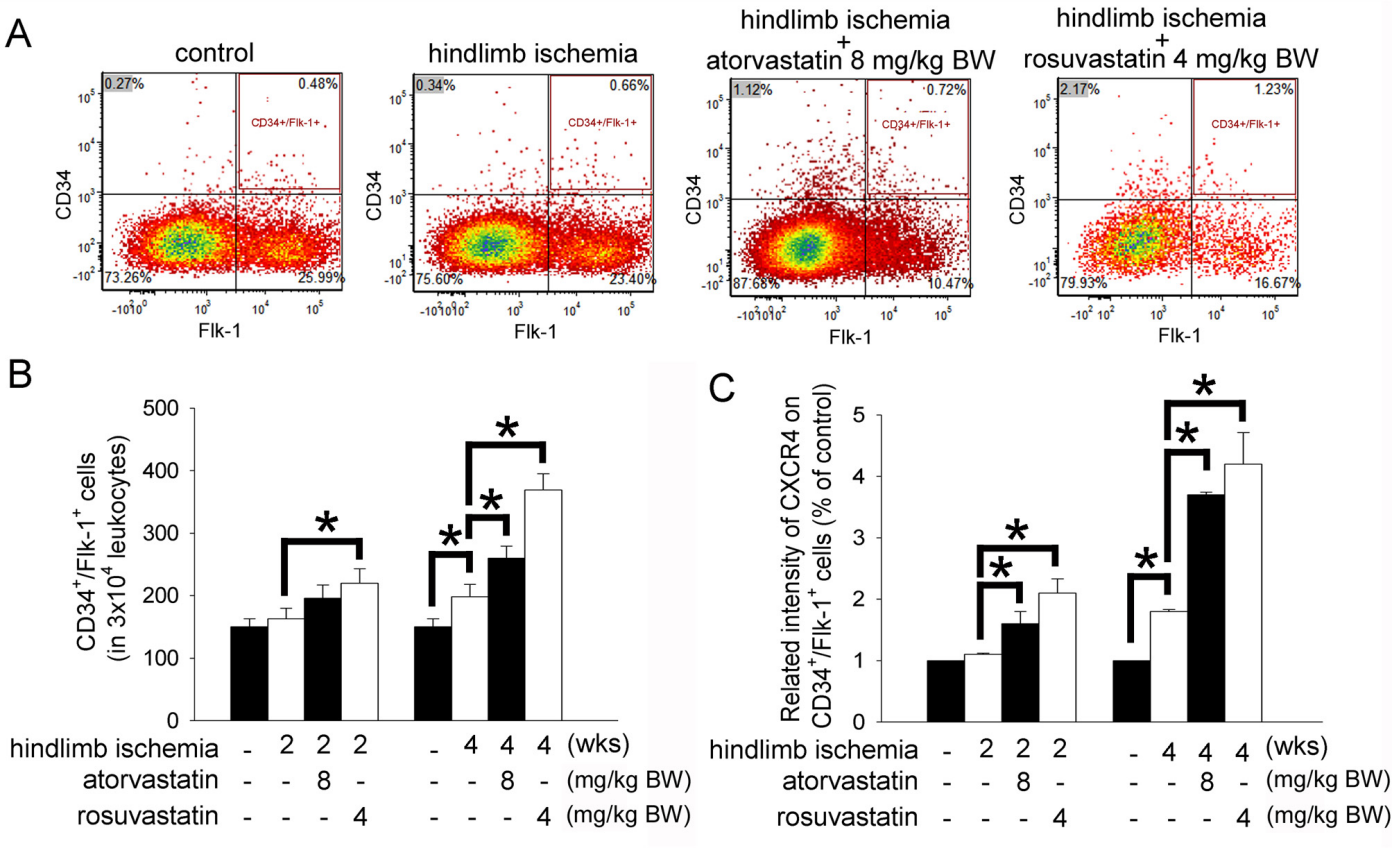
The effects of atorvastatin and rosuvastatin on CXCR4 expression on circulating EPCs in hindlimb ischemic mice. (A) EPC (defined as CD34^+^/Flk-1^+^ cells) mobilization after hindlimb ischemia surgery for 4 weeks was determined using flow cytometry. (B) The graph showed the quantification of CD34^+^/Flk-1^+^ cells in hindlimb ischemic and statin-administered mice. (C) The graph shows the quantification CXCR4 signaling density on CD34^+^/Flk-1^+^ cells in hindlimb ischemic and statin-administered mice. The results are expressed as the mean ± SEM (n = 5, *p < 0.05 was considered to be significant).

We further wanted to analyze whether the statins treatment increased the number of EPCs in the blood of ischemic mice. After hindlimb ischemia surgery, the populations of endogenous EPCs (defined as CD34^+^/Flk-1^+^ cells) were quantified by flow cytometry (Fig 4A). The isotype antibody control was presented in the S2 Fig. At the 2^nd^ week, ischemic mice from the 4 mg/kg BW rosuvastatin-treated but not the 4 mg/kg BW atorvastatin-treated group exhibited significantly increased numbers of mobilized EPCs in the peripheral blood compared with the untreated group. Until the 4^th^ week, the 8 mg/kg BW atorvastatin-treated groups exhibited significantly increased numbers of mobilized EPCs in the peripheral blood compared with the controls (Fig 4B). Additionally, we analyzed CXCR4 expression on the circulating EPCs. Our results reveled that hindlimb ischemia surgery may have induced more intense CXCR4 expression on circulating CD34^+^/Flk-1^+^ EPCs; treatment with either atorvastatin or rosuvastatin may have significantly up-regulated the relative intensity of CXCR4 expression on CD34^+^/Flk-1^+^ EPCs (Fig 4C). Accordingly, our findings demonstrated that both atorvastatin and rosuvastatin could elevate the numbers of circulating EPCs, as well as up-regulate the expression of CXCR4 on EPCs in the blood.

### Atorvastatin and rosuvastatin effectively promoted the neovasculogenesis of human EPCs

**Fig 5:**
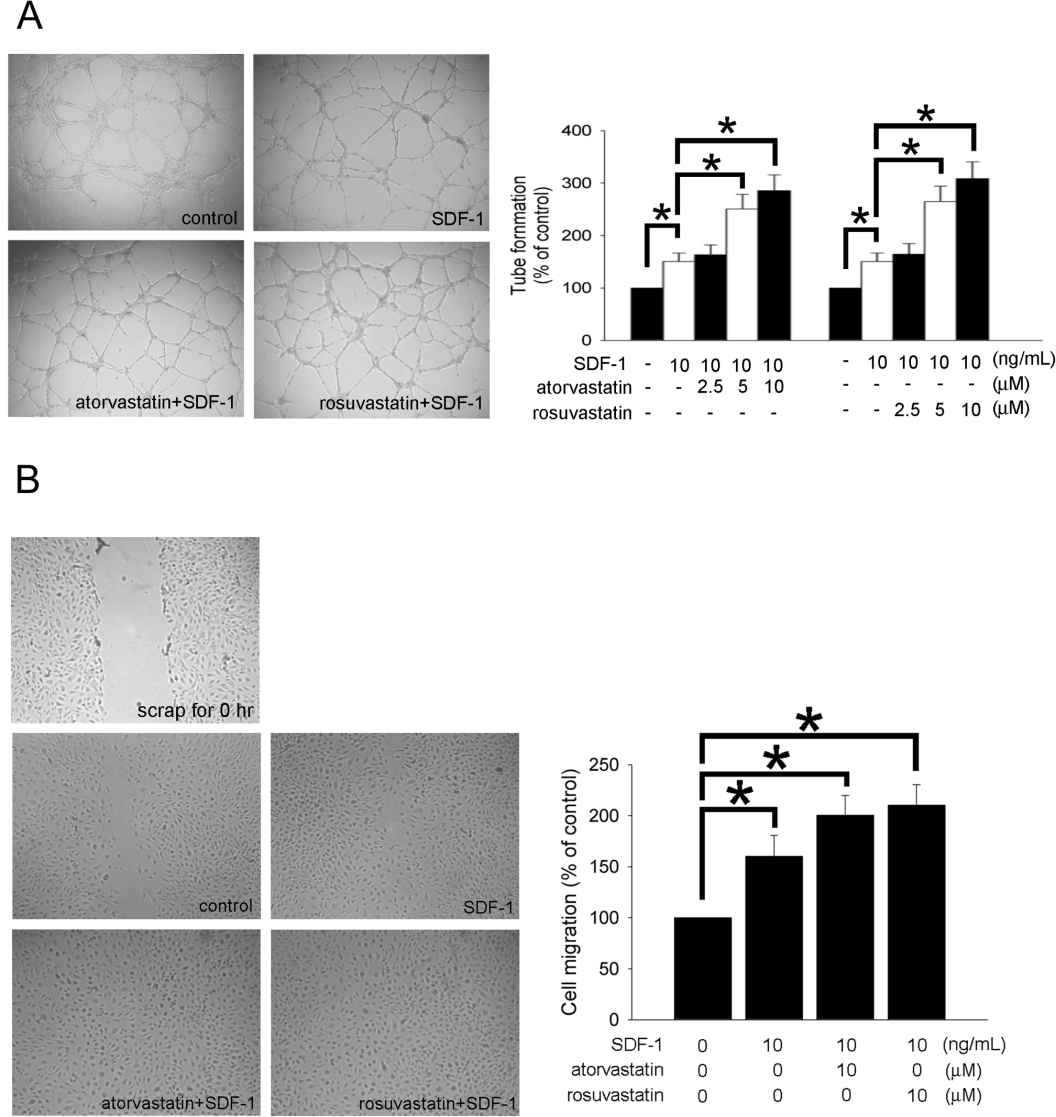
The effects of atorvastatin and rosuvastatin on human EPC functions. (A) After treating EPCs with 2.5–10 μM atorvastatin or rosuvastatin for 24 hours, an *in vitro* angiogenesis assay using the ECMatrix gel with or without 10 ng/mL recombinant human SDF-1 was conducted to investigate the effects of statins on EPC neovascularization. Representative photos of *in vitro* angiogenesis are shown. The graph shows the quantification of tube formation by statin-treated EPCs following SDF-1 administration. (B) A wound-healing assay was used to evaluate the effect of statins on the migration of SDF-1-treated EPCs. EPCs were cultured with 10 μM atorvastatin or rosuvastatin for 24 hours before wound scraping was performed using a pipette tip. Then, the EPCs were cultured in the presence of 10 ng/mL SDF-1, and images were taken 8 hours after wound scraping. EPCs that migrated to the denuded area were counted based on the black baseline. EPCs that migrated into the denuded area were quantified, and the magnitude of EPC migration was evaluated by counting the migrated cells in six random areas under high-power microscopic fields (×100). Data are expressed as the mean ± SEM of three independent experiments and as the percentage of the control (n = 5, *p < 0.05 was considered to be significant).

To clarify whether atorvastatin and rosuvastatin is involved in EPC-mediated neovasculogenesis, the *in vitro* tube formation assay and wound-healing assay were used. The effects of statins on cell cytotoxicity were analyzed by MTT assay (S1 Fig) After 24 hours treatment with 2.5–10 μM of atorvastatin or rosuvastatin, the tube-forming capacity of the statins-treated EPCs in the presence of SDF-1 was significantly up-regulated compared with that of the controls (Fig 5A). In addition, the wound-healing assay was used to evaluate the effect of statins on the migration of SDF-1-treated EPCs. First, human EPCs were pre-treated with 10 μM atorvastatin or rosuvastatin for 24 hours before wound scraping. After that, the EPCs were cultured in the presence of 10 ng/mL SDF-1, and images were taken 8 hours after wound scraping. Next, EPCs that migrated to the denuded area were counted. Both the atorvastatin and rosuvastatin treatments significantly accelerated wound closure compared with the controls (Fig 5B). The results showed that atorvastatin and rosuvastatin effectively promoted the neovasculogenesis ability of EPCs.

### The eNOS-related pathways may contribute to CXCR4 expression on atorvastatin and rosuvastatin-administered EPCs

**Fig 6:**
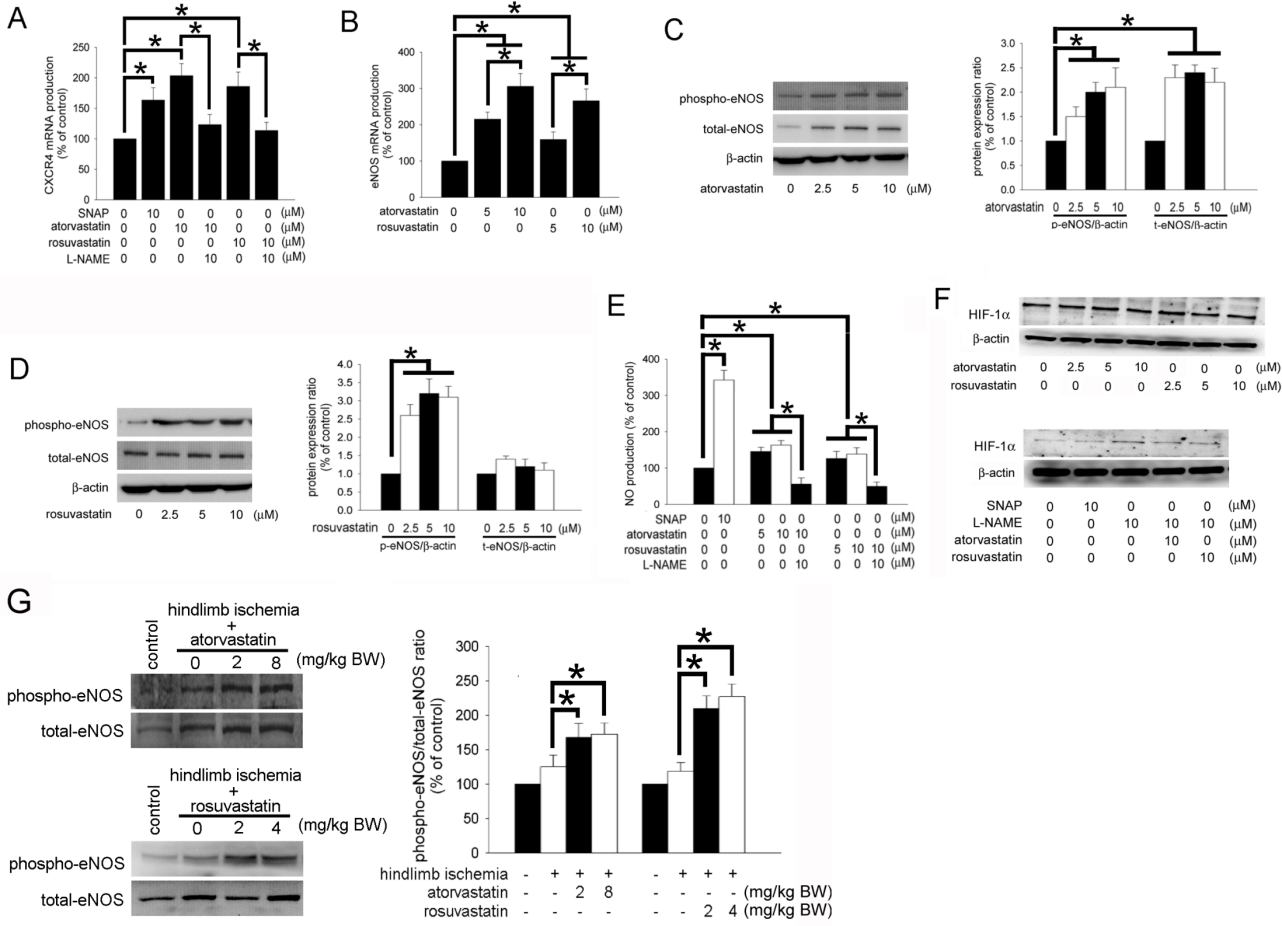
The eNOS-related pathways may contribute to CXCR4 expression on statin-treated EPCs. (A) EPCs were treated with 10 μM atorvastatin or rosuvastatin for 24 hours. CXCR4 mRNA levels were quantified using quantitative real-time PCR. Additionally, SNAP and L-NAME treatment for 1 hour prior to the statin treatment was used to identify the involvement of NO-mediated signaling in CXCR4 expression. (B) EPCs were treated with 5–10 μM atorvastatin or rosuvastatin for 12 hours. eNOS mRNA levels were quantified using quantitative real-time PCR. (C and D) EPCs were treated with 2.5–10 μM atorvastatin or rosuvastatin for 12 hours. The amount of total eNOS and phospho-eNOS were analyzed by western blotting. β-actin protein levels were used as a loading control. The graph shows the quantification of protein expression (total eNOS/β-actin and phospho-eNOS/β-actin ratios). (E) EPCs were treated with or without SNAP and L-NAME for 1 hour prior to 5–10 μM statin treatment for 18 hours. NO levels in the cultured medium were analyzed using ESR. (F) Upper: EPCs were treated with 2.5–10 μM atorvastatin or rosuvastatin for 12 hours. The HIF-1α was analyzed by western blotting. The β-actin protein levels were used as a loading control. Lower: EPCs were treated with or without L-NAME for 1 hour prior to 10 μM statin treatment, or SNAP treatment only for 12 hours. HIF-1α was analyzed by western blotting. (G) Phospho-eNOS and total eNOS protein levels in the ischemic muscle were analyzed by western blotting analysis. The phospho-eNOS/total eNOS ratio is shown as a bar graph. Data are expressed as the mean ± SEM of three independent experiments and as the percentage of the control. (n = 5, *p < 0.05 was considered to be significant).

In animal studies, we found that both atorvastatin and rosuvastatin treatment up-regulated the expression of CXCR4 on the EPCs. Previous evidence had demonstrated that eNOS plays critical roles in the functions of EPCs. Therefore, we wanted to analyze whether eNOS activity was also involved in this issue. S-nitroso-N-acetyl-penicillamine (SNAP, an NO donor) and *N*-nitro-l-arginine methyl ester (L-NAME, an NO synthase inhibitor) were used 1 hour prior to the statins treatment to identify whether NO-mediated signaling pathways were involved in CXCR4 expression. Indeed, when human EPCs were pretreated with SNAP, CXCR4 mRNA expression was significantly up-regulated. In contrast, after pretreatment with L-NAME, the induction of CXCR4 mRNA expression by the statins treatment was significantly reduced (Fig 6A). Subsequently, human EPCs were treated with 5–10 μM atorvastatin or rosuvastatin for 12 hours, and the production of eNOS mRNA were quantified by using qPCR. In cells treated with either atorvastatin or rosuvastatin, the amount of eNOS mRNA was significantly increased (Fig 6B). Human EPCs were treated with 2.5–10 μM atorvastatin or rosuvastatin for 12 hours, and the eNOS activity was analyzed by western blotting analysis. Upon treating human EPCs with 2.5–10 μM atorvastatin, both phospho-eNOS protein and total eNOS protein levels were significantly increased (Fig 6C). In contrast, in human EPCs treated with 2.5–10 μM rosuvastatin, phospho-eNOS but not total eNOS protein levels were significantly induced (Fig 6D). Subsequently, the amount of NO in EPC-cultured medium was analyzed after atorvastatin and rosuvastatin treatments. First, human EPCs were treated with or without SNAP and L-NAME for 1 hour prior to 5–10 μM statin treatment for 18 hours, and the NO production by EPCs was detected in the cultured medium by the NO spin-trapping technique using electron spin resonance (ESR) spectroscopy. Both atorvastatin and rosuvastatin appreciably stimulated NO production and release into the culture medium. On the contrary, the statins-induced NO production was significantly reduced after pre-treatment with L-NAME (Fig 6E). NO restores HIF-1α hydroxylation during hypoxia. Even though atorvastatin and rosuvastatin increased the activation of NO, treatment of atorvastatin and rosuvastatin only did not affect the accumulation of HIF-1α. However, treated with L-NAME may increase the HIF-1α expression, which may reverse by atorvastatin and rosuvastatin treatment. Additionally, the phospho-eNOS and total eNOS proteins levels in ischemic hindlimb muscle were also detected by western blotting analysis. The phospho-eNOS protein level was significantly induced after treatment with 2 and 8 mg/kg BW atorvastatin or 2 and 4 mg/kg BW rosuvastatin (Fig 6G). Above all, this suggests that the NO signaling pathway participates in CXCR4 expression in atorvastatin and rosuvastatin-treated EPCs.

## Discussion

In our study, we demonstrated that the most potent statins atorvastatin and rosuvastatin promoted small vessel formation, which enhances the recovery of capillary density in ischemic hindlimb mice. Here, we show that both atorvastatin and rosuvastatin encouraged EPC-mediated neovascularization by *in vitro* and *in vivo* analyses. Atorvastatin and rosuvastatin treatments increased the numbers of CXCR4-positive EPCs in the blood and recruited to the ischemia tissues. By in vitro assays, we showed that both atorvastatin and rosuvastatin led to up-regulated CXCR4 mRNA expression in human EPCs, as well as d the functions of human EPCs, including their tube formation and migration abilities. Our results showed that both atorvastatin and rosuvastatin regulated EPC neovascularization, possibly through the contribution of eNOS-related pathways to the up-regulation of CXCR4 in EPCs.

The effect of the statins family on neovasculogenesis has been demonstrated both *in vivo* and *in vitro*, but accurate mechanisms by which atorvastatin or rosuvastatin act in the ischemic tissue are currently not clear. Additionally, statins have been shown to enhance neovasculogenesis by activating the endothelial PI-3 kinase/Akt/eNOS/NO pathway and up-regulating eNOS expression [20,21]. Following statin administration, several genes are activated in the ischemic tissue that stimulate ischemic neovasculogenesis by promoting the homing of EPCs [22]. In addition, the SDF-1/CXCR4 axis has been implicated in the regulation of inflammation and angiogenesis. However, the role of CXCR4 in EPC homing, engraftment and neovascularization following statins treatment has not been fully clarified. In our study, we demonstrated that atorvastatin or rosuvastatin could significantly enhance EPC mobilization and recruitment to the ischemic tissue for neovascularization. First, we demonstrated that both atorvastatin and rosuvastatin promoted vasculogenesis and the up-regulation of CXCR4 expression in the ischemic tissue.

Previous studies showed that EPCs could be homed to sites of ischemia, where they play a pivotal role in adult neovascularization and repair of the damaged endothelium. [23,24]. The pleiotropic effects of statins on the circulating number and functional activity of EPCs have been studied. The cytokine/chemokine gradient is the key factor that promotes EPC migration from the bone marrow niche to sites of neovasculogenesis [25]. Additionally, EPCs migrate toward the angiogenic gradient via chemokine receptors such as CXCR4 and VEGFR-2 [25]. A recent study indicated that statins could activate the CCR7 emigration pathway in macrophages to accelerate the regression of atherosclerosis [26]. However, the mechanism by which rosuvastatin or atorvastatin improved the neovasculogenesis of EPCs remained unclear. The major observation of our study is that both atorvastatin and rosuvastatin treatments enhance the response of EPCs to ischemic tissue, inducing a significant increase in the number of circulating EPCs that co-express the homing receptor CXCR4. In our *in vitro* assays, atorvastatin or rosuvastatin up-regulated CXCR4 mRNA expression and enhanced the mobilization of EPCs. These findings suggest that statins accelerate vasculogenesis after ischemia via the SDF-1/CXCR4 axis.

Two different mechanisms, vasculogenesis and angiogenesis involve in the formation of the vascular network in the embryo and adult. Vasculogenesis is the process of blood vessel formation occurring by a de novo production of endothelial cells. However, angiogenesis is the physiological process through which new blood vessels form from pre-existing vessels and expansion of vessel network. Vasculogenesis and angiogenesis are normal and vital processes in growth of individual and development of diseases. Even though the atorvastatin or rosuvastatin improve neovascularization by increasing the expression density of CXCR4 in endothelial progenitor cells, as well as increasing EPC-mediated recovery of capillary density and blood flow *in vivo*, we also can not exclude the effects of statin in angiogenesis (mature vessel formation, sprouting angiogenesis and intussusceptive angiogenesis)(S3 Fig). Therefore, it is necessary to explore the mechanisms involving in statin-induced angiogenesis in the further.

Several studies have established the role of eNOS in maintaining physiological cardiovascular function and preventing cardiovascular pathophysiology [27]. Statins exert their beneficial effects by increasing eNOS expression and activity during the processes of atherosclerosis and endothelial dysfunction [27]. Rosuvastatin treatment in apoE-knockout dyslipidemic mice decreased caveolin-1 expression and promoted eNOS function, along with concurrent improvements in blood pressure and heart rate variability [28]. Atherosclerosis in moderately hypercholesterolemic rabbits induced by L-NAME, a NOS inhibitor, is suppressed by pitavastatin via the inhibition of macrophage accumulation and foam cell formation [29]. Atorvastatin conferred significant protection against ischemia-reperfusion injury in rats fed a high-fructose diet by increasing NOS expression through an Akt-dependent pathway [27]. In our study, we demonstrated that atorvastatin or rosuvastatin activated the eNOS signaling pathway and up-regulated CXCR4 expression both in the ischemic tissue and in EPCs.

Our study showed for the first time that atorvastatin or rosuvastatin could potentially increase CXCR4 expression in ischemic tissue and EPCs, thereby promoting EPC homing to ischemic tissues and neovascularization.

## Limitation

Even though our evidences demonstrated that the efficacy of atorvastatin and rosuvastatin on improving EPC neovascularization was related to the SDF-1α/CXCR4 axis and might be regulated by the NO, there was rare evidences to provide the molecular mechanism of how atorvastatin and rosuvastatin increase expression of CXCR4 in EPCs. We know that is very important, and readers are also highly interested in the issue. Additionally, it seems to be rather difficult to clarify this issue in the current relevant animal experiments, and it is the major limitation in this study.

## Supporting Information

S1 Fig Method for cell cytotoxicity assay. The effects of atorvastatin and rosuvastatin on cell cytotoxicity were analyzed by the 3-(4,5-dimethylthiazol-2-yl) -2,5-diphenyl tetrazolium bromide (MTT) assay. Late EPCs (2x10^4^ cells) were grown in 96-well plates and incubated with 2.5–20 μM statins for 6–24 h. Subsequently, MTT (0.5 μg/mL) was applied to cells for 4 h. The cells were lysed with dimethylsulfoxide (DMSO), and the absorbance was read at 530 nm using a DIAS Microplate Reader (Dynex Technologies, Chantilly, VA, USA). The effects of atorvastatin and rosuvastatin on cell cytotoxicity. (A and B) After treatment of EPCs with 2.5–20 μM of atorvastatin or rosuvastatin for 6–24 hours, the cell cytotoxicity of statin was analysis using MTT assay. Data were expressed as the mean ± SEM of three experiments performed in triplicate.*p < 0.05 was considered significant which compare to control group at the same time point.
(TIF)

S2 Fig Isotype controls for identification of EPCs. Peripheral blood was incubated with FITC conjugated anti-mouse CD34 (eBioscience, San Diego, CA, USA), PE conjugated anti-mouse Flk-1 (VEGFR-2, eBioscience, San Diego, CA, USA), or APC conjugated anti-mouse CXCR4 (Becton Dickinson, San Jose, CA, USA) antibodies. Isotype-identical antibodies served as controls (Becton Dickinson, Franklin Lakes, NJ, USA). The expression of fluorescence was observed using flowcytometry. The results suggested that these three kinds of antibodies may efficiency to identify the CD34, Flk-1 and CXCR4 expression on the cell surface.
(TIF)

S3 Fig Statins increased the formation of mature vessels in the ischemic muscle. The effects of atorvastatin and rosuvastatin on mature vessel formation in the skeletal muscle after hindlimb ischemia in ICR mice. Mice were sacrificed 4 weeks after surgery, and the expression of α-SMA and CD31 in the ischemic muscles were visualized by immunostaining (original magnification x400), respectively. The α-SMA and CD31 are indicated with white arrows. Hoechst stain was used to counterstain the nucleus. The results indicated that atorvastatin or rosuvastatin administration significantly increased the formation of mature vessels in the ischemic muscle compared with that in the non-statin treatment group.
(TIF)
