# Supplementary figures and images for: Statins, HMG-CoA Reductase Inhibitors, Improve Neovascularization by Increasing the Expression Density of CXCR4 in Endothelial Progenitor Cells
Source: PLoS One. 2015 Aug 26;10(8):e0136405. doi: 10.1371/journal.pone.0136405 (PMC4550447; doi:10.1371/journal.pone.0136405)

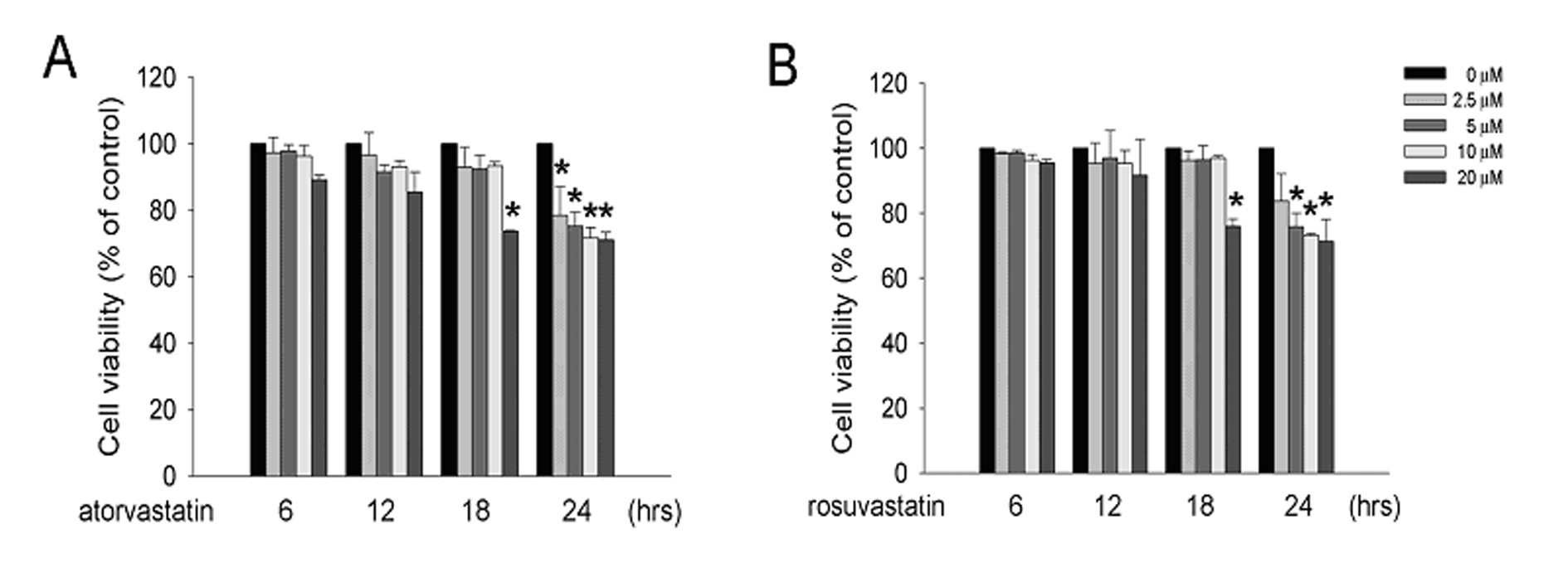

Supplement: S1 Fig — The effects of atorvastatin and rosuvastatin on cell cytotoxicity were analyzed by the 3-(4,5-dimethylthiazol-2-yl) -2,5-diphenyl tetrazolium bromide (MTT) assay. Late EPCs (2x10^4^ cells) were grown in 96-well plates and incubated with 2.5–20 μM statins for 6–24 h. Subsequently, MTT (0.5 μg/mL) was applied to cells for 4 h. The cells were lysed with dimethylsulfoxide (DMSO), and the absorbance was read at 530 nm using a DIAS Microplate Reader (Dynex Technologies, Chantilly, VA, USA). The effects of atorvastatin and rosuvastatin on cell cytotoxicity. (A and B) After treatment of EPCs with 2.5–20 μM of atorvastatin or rosuvastatin for 6–24 hours, the cell cytotoxicity of statin was analysis using MTT assay. Data were expressed as the mean ± SEM of three experiments performed in triplicate.*p < 0.05 was considered significant which compare to control group at the same time point. (TIF) [file pone.0136405.s001.tif]

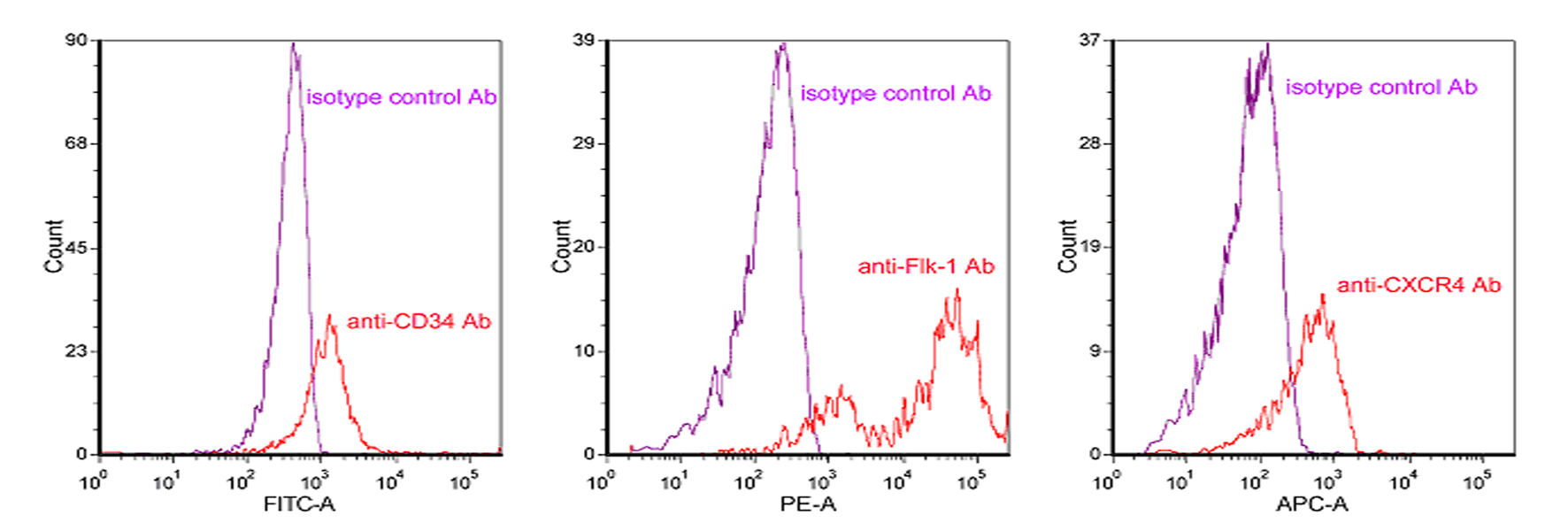

Supplement: S2 Fig — Peripheral blood was incubated with FITC conjugated anti-mouse CD34 (eBioscience, San Diego, CA, USA), PE conjugated anti-mouse Flk-1 (VEGFR-2, eBioscience, San Diego, CA, USA), or APC conjugated anti-mouse CXCR4 (Becton Dickinson, San Jose, CA, USA) antibodies. Isotype-identical antibodies served as controls (Becton Dickinson, Franklin Lakes, NJ, USA). The expression of fluorescence was observed using flowcytometry. The results suggested that these three kinds of antibodies may efficiency to identify the CD34, Flk-1 and CXCR4 expression on the cell surface. (TIF) [file pone.0136405.s002.tif]

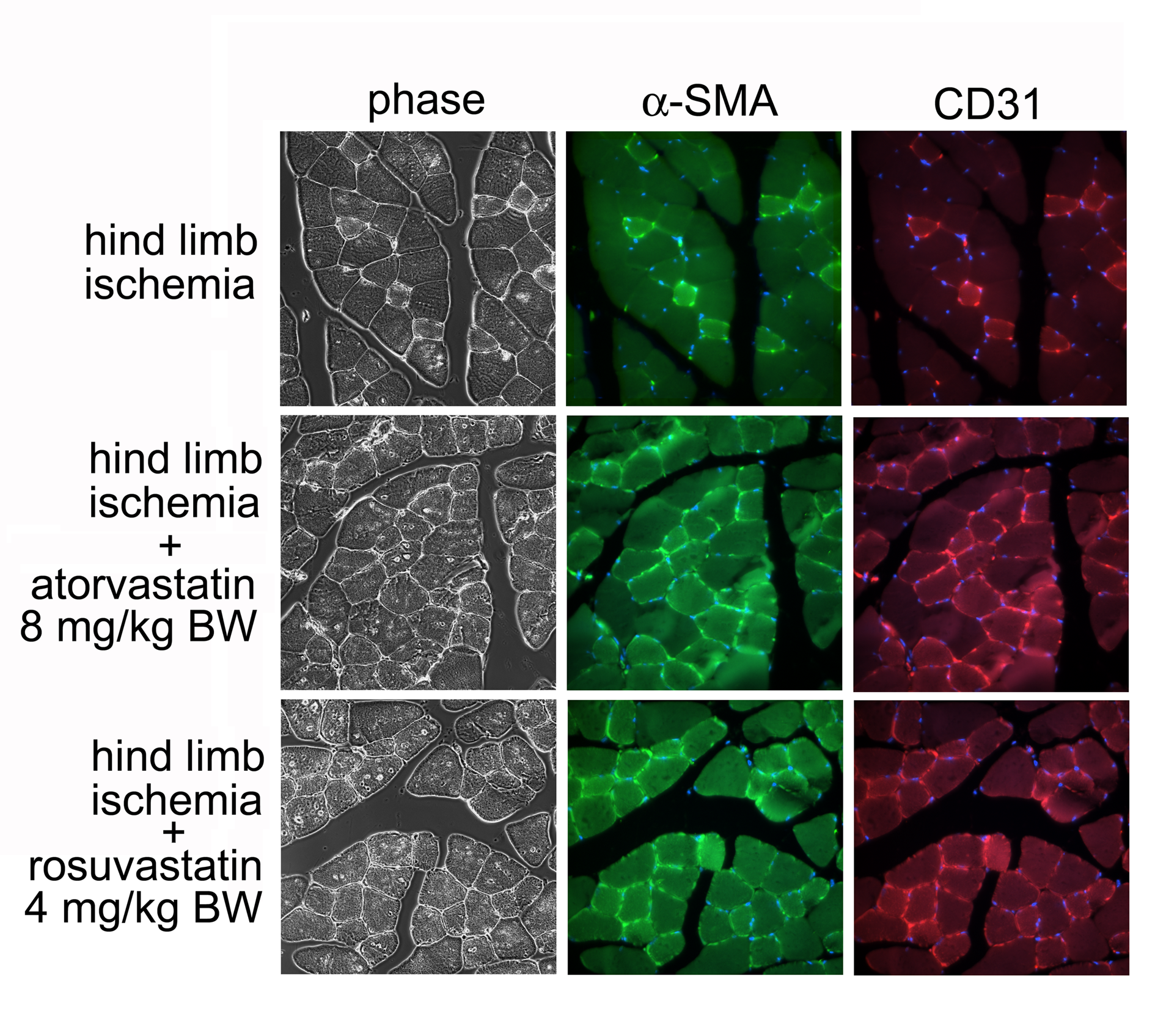

Supplement: S3 Fig — The effects of atorvastatin and rosuvastatin on mature vessel formation in the skeletal muscle after hindlimb ischemia in ICR mice. Mice were sacrificed 4 weeks after surgery, and the expression of α-SMA and CD31 in the ischemic muscles were visualized by immunostaining (original magnification x400), respectively. The α-SMA and CD31 are indicated with white arrows. Hoechst stain was used to counterstain the nucleus. The results indicated that atorvastatin or rosuvastatin administration significantly increased the formation of mature vessels in the ischemic muscle compared with that in the non-statin treatment group. (TIF) [file pone.0136405.s003.tif]
